# Editorial: Novel technologies in the diagnosis and management of sleep-disordered breathing, volume II

**DOI:** 10.3389/frsle.2025.1696478

**Published:** 2025-10-10

**Authors:** Ding Zou, Henri Korkalainen

**Affiliations:** ^1^Center for Sleep and Vigilance Disorders, Institute of Medicine, Sahlgrenska Academy, University of Gothenburg, Gothenburg, Sweden; ^2^Department of Technical Physics, University of Eastern Finland, Kuopio, Finland; ^3^Diagnostic Imaging Center, Kuopio University Hospital, Kuopio, Finland

**Keywords:** obstructive sleep apnea, digital pathway, precision medicine, biomarker, Mendelian randomization, comorbidity, photoplethymograph (PPG), sleep health

Volume II of our *Frontiers* Research Topic Series was completed in September 2024. Although nearly a year passed before we began drafting the editorial, this delay proved advantageous. The intervening period witnessed notable advances in the field of sleep-disordered breathing (SDB), allowing us to reflect not only on the contributions of this volume but also on the rapidly evolving scientific landscape in which these studies are situated. For example, key regulatory milestones were achieved, including the approval of the first pharmacological treatment for obese patients with obstructive sleep apnea (OSA) by the U.S. Food and Drug Administration (Lisik and Zou, 2025). The American Academy of Sleep Medicine also released guidelines for the treatment of central sleep apnea, underscoring the dynamic and evidence-based evolution of clinical practice (Badr et al., 2025). Furthermore, projections indicate that by 2050 nearly 77 million U.S. adults aged 30–69 will be living with OSA, a 35% increase compared with 2020 (Boers et al., 2025). The global health and economic costs are enormous: health systems are strained by diagnosis delays (overnight polysomnography is resource-intensive) and low adherence to conventional therapies (e.g. CPAP). These challenges have motivated the development of novel diagnostic and management technologies for SDB and the recent developments provide important context for the diverse contributions featured in this volume.

## Genetic insights

Genetic research has long contributed to our understanding of SDB, from early candidate gene studies implicating pathways related to obesity, craniofacial development, and ventilatory control, to more recent genome-wide association studies. Within this broader context, Mendelian randomization (MR) has emerged as a methodological framework that leverages genetic variation to make causal inferences about the relationship between exposures and outcomes. MR is often described as a form of “nature's randomized trial,” because the random allocation of alleles during meiosis approximates the randomization process used in clinical trials (Smith and Ebrahim, 2003). This approach has gained traction in sleep research because it helps disentangle correlation from causation, a particularly pressing challenge given the multifactorial nature of OSA and its numerous comorbidities.

Three studies in this volume applied MR to examine associations between OSA and diverse outcomes. Gong et al. utilized the GSE135917 OSA gene dataset, derived from subcutaneous adipose tissue samples of OSA patients. Through weighted gene co-expression network analysis, they identified two critical genes, CETN3 and GTF2A2, that may contribute to OSA pathogenesis. These findings suggest new molecular targets for further investigation, potentially paving the way toward biomarker-driven precision medicine. Yang et al., drawing on data from the National Health and Nutrition Examination Survey (NHANES), reported a causal link between OSA and increased risk of osteoarthritis, with body mass index serving as a mediator. This highlights the intertwined relationship between SDB, obesity, and musculoskeletal health, and suggests that comprehensive management of OSA may also confer benefits beyond sleep, particularly in reducing osteoarthritis burden. In contrast, Hou et al. analyzed data from the FinnGen database and found no causal evidence that genetically predicted OSA leads to chronic kidney disease, contradicting the findings from observational studies. However, higher blood urea nitrogen (BUN; a marker of renal dysfunction) predicted increased OSA risk. This suggests that renal impairment may exacerbate OSA or share common pathways, but specific pathways remain elusive. Although negative findings often receive less attention, this helps refine hypotheses and redirect future efforts.

Together, these MR studies emphasize the complexity of OSA as both a consequence of genetic predisposition and a risk factor for systemic disease. However, the rise of MR has not been without controversy. The method depends on strong assumptions: namely, that the selected genetic variants influence the outcome only through the exposure of interest, are not linked to other pathways (no horizontal pleiotropy), and are robustly associated with the exposure. When these conditions are not met, results can be biased and misleading (Evans et al., 2025). Given the methodological intricacies of MR, it is recommended that future investigations adhere to the MR-SLEEP guidelines, which provide a structured framework for robust study design and interpretation (Evans et al., 2025).

## Epidemiology, comorbidities, and the burden of OSA

Several contributions in this volume highlight the global prevalence of SDB and its far-reaching clinical consequences. Wang et al. reported striking differences in SDB prevalence among patients with multiple system atrophy, documenting a prevalence of 79% in an Asian cohort compared with 42% reported in European populations. These findings raise important questions about genetic, environmental, and diagnostic factors that may account for the disparity. Moreover, the results highlight the need for efficient screening in populations with a high likelihood of SDB.

The interplay between OSA and infectious disease was examined by Dinh et al., who demonstrated that moderate-to-high risk of OSA is associated with more severe manifestations of COVID-19. The authors postulated that dysregulated inflammatory responses in OSA patients contribute to worse outcomes, reinforcing the concept that sleep health is integrally tied to immune function. Mental health comorbidities were also explored. Li et al. applied latent profile analysis to depressive symptoms in NHANES participants with OSA symptoms, identifying distinct clusters in the population, illustrating the heterogeneity of psychological burden in OSA patients. Collectively, these studies reinforce the urgent need to improve diagnostic pathways for OSA and to acknowledge its systemic consequences.

In line with these findings, Pittman et al. convened a group of experts to critically evaluate the challenges of OSA management facing in the United States. They identified five domains requiring urgent reform: (1) simplify the patient journey, (2) bridge the communication gaps, (3) expand the monitoring over several nights for serial assessments and therapy titration, (4) update the care models to avoid provider shortages and burnout, and (5) align the financial models to reward high-quality care. Their work represents a call to action for structural change and highlights the necessity of aligning healthcare delivery with the realities of OSA's widespread burden.

## Mechanistic and biomarker studies

To advance mechanistic understanding of OSA and explore novel biomarkers, Howarth et al. investigated electroencephalogram (EEG) power spectral densities in patients with mild OSA. They reported a positive association between relative delta frequency power and excessive daytime sleepiness as assessed by the multiple sleep latency test. These results suggest that mild OSA patients with elevated delta activity may experience increased sleep drive, a finding that raises conceptual questions about how to distinguish excessive sleepiness from excessive need for sleep. Biomarker discovery was advanced by Liu et al., who conducted the first study of claudin (CLDN) proteins, key regulators of barrier function, in OSA. Their analysis revealed that plasma and urine CLDN levels decreased in OSA patients, and especially urinary CLDN3 was inversely associated with OSA severity. These findings are intriguing, as they suggest a possible mechanistic link between intermittent hypoxia, intestinal mucosal integrity, and altered biomarker expression. Future studies are needed to determine whether disrupted epithelial barriers contribute to systemic consequences of OSA.

## Cardiovascular insights and heart rate dynamics

Cardiovascular complications represent one of the most significant consequences of OSA. While physiological signals representing the cardiovascular status are routinely measured during sleep recordings, they often remain underutilized. Heart rate, for example, has traditionally been viewed as supplementary to respiratory or oxygenation metrics in sleep studies. However, emerging research underscores its value as a sensitive marker of autonomic and cardiovascular stress. Hilmisson et al. employed photoplethysmography (PPG) based cardiopulmonary coupling analysis (Thomas et al., 2005) to derive heart rate measures during sleep. They reported that each one beat per minute increase during stable non-REM sleep corresponded to a 4.4% higher likelihood of nocturnal non-dipping blood pressure, an established cardiovascular risk factor. These findings support the proposition that nocturnal heart rate dynamics may serve as accessible and clinically relevant markers for cardiovascular health assessment in OSA patients (Azarbarzin et al., 2025).

## Technological innovations and protocol development

Innovation in therapeutic strategies and methodological rigor is also reflected in this volume. O'Connor Reina et al. clarified the development of a smartphone-based application for myofunctional therapy, designed to strengthen pharyngeal dilator muscle control in OSA patients. This work reflects the broader trend toward digital therapeutics, which offer scalability and accessibility for tailored therapy.

Additionally, Kobayashi Frisk et al. presented a systematic review protocol aimed at synthesizing evidence on multidimensional sleep health (Buysse, 2014) and cardiovascular disease. Protocol publications are valuable for enhancing transparency and reducing bias, and this initiative signals a growing recognition of the need to move beyond unidimensional measures of sleep (e.g., sleep duration) toward more holistic conceptualizations.

## Future directions in OSA management

Taken together, the studies in this volume underscore that the field of sleep medicine is progressing rapidly, both in diagnostic precision and therapeutic innovation. Yet, realizing the benefits of these advancements will require systemic transformation. The World Health Organization's recent report envisions care pathways that integrate home sleep apnea testing, telemonitoring, and virtual consultations to accelerate access and tailor management (WHO, 2025). Such pathways not only streamline diagnosis and treatment but also hold promise for improving patient outcomes by reducing delays and increasing personalization (Zou et al., 2023).

At the same time, the integration of machine learning, advanced diagnostic modalities, and novel therapies heralds a new era of personalized medicine in sleep care. However, successful translation of advanced diagnostics and personalized treatments from research into clinical practice will require careful validation in large-scale, diverse populations (McNicholas and Korkalainen, 2023; Oks et al., 2025). Moreover, innovations must be accompanied by health system reforms to ensure equitable access, particularly for underserved populations disproportionately affected by OSA. Equally critical is the training of healthcare professionals as new diagnostic technologies, digital health platforms, and personalized therapeutic strategies will necessitate continuous education and interdisciplinary collaboration (McNicholas et al., 2025a). Sleep medicine increasingly intersects with respirology, cardiology, endocrinology, neurology, and psychiatry, reinforcing the need for collaborative approaches that transcend traditional disciplinary boundaries. Finally, addressing the systemic burden of OSA requires not only technological and clinical innovation but also policy-level interventions (McNicholas et al., 2025b). Financial models must evolve to reward high-quality, patient-centered care, and workforce challenges must be mitigated through strategic planning and resource allocation.

## Conclusion

Volume II of our *Frontiers* Research Topic series captures a pivotal moment in sleep medicine, showcasing studies that range from molecular genetics to health policy. Together, these contributions reinforce the view of OSA as a complex, multifactorial disorder with implications across nearly every organ system. At the same time, they illuminate new avenues for diagnosis, treatment, and health system reform. The field stands at the threshold of a transformative era, and the challenge ahead is to translate these scientific advances into tangible benefits for patients worldwide (Kryger and Thomas, 2025).
